# The Value of the ALBI and ALBI–PLT Scores in Predicting Esophageal Varices in Cirrhotic Patients: A Cross‐Sectional Study

**DOI:** 10.1002/hsr2.72892

**Published:** 2026-07-25

**Authors:** Thang Dinh Nguyen, Huong Tu Lam, Thong Duy Vo

**Affiliations:** ^1^ Department of Gastroenterology Cho Ray Hospital Ho Chi Minh City Vietnam; ^2^ Department of Internal Medicine, School of Medicine University of Medicine and Pharmacy at Ho Chi Minh City Ho Chi Minh City Vietnam; ^3^ Department of Gastroenterology University Medical Center Ho Chi Minh City Ho Chi Minh City Vietnam

## Abstract

**Background and Aims:**

Esophageal varices (EVs) are a common and life‐threatening complication of liver cirrhosis. Endoscopic screening is recommended but remains invasive and resource‐intensive. This study aimed to evaluate the diagnostic performance of the albumin–bilirubin (ALBI) and ALBI–platelet (ALBI–PLT) scores for predicting the presence of esophageal varices in cirrhotic patients.

**Methods:**

This cross‐sectional study included 152 patients with liver cirrhosis who underwent screening endoscopy at Cho Ray Hospital between November 2022 and May 2023. ALBI and ALBI–PLT scores were calculated from routine laboratory parameters. Receiver operating characteristic (ROC) analysis was performed to evaluate diagnostic performance.

**Results:**

Among 152 cirrhotic patients (mean age 56 years, 65.8% male), the prevalence of EVs was 56%. The ALBI score showed moderate predictive performance for EVs (AUC 0.78; 95% CI 0.70–0.84) and varices needing treatment (AUC 0.79). The ALBI–PLT score demonstrated similar performance (AUC 0.76 and 0.77, respectively). Using a cutoff of ALBI > −2.76, 20.4% of patients could potentially avoid screening endoscopy, with 9.7% of varices needing treatment missed.

**Conclusion:**

ALBI and ALBI–PLT scores may serve as simple non‐invasive triage tools to identify low‐risk cirrhotic patients who may safely defer screening endoscopy, particularly in resource‐limited settings.

**Abbreviations**
ALBI: albumin‐bilirubin
ALBI‐PLT: albumin‐bilirubin‐platelets
AUC: area under the curve
CI: confidence interval
CTP: Child‐Pugh
EVs: esophageal varices
HRVs: high risk esophageal varices
HVPG: hepatic venous pressure gradient
NPV: negative predictive value
PHTN: portal hypertension
PPV: positive predictive value

## 1 Introduction

Cirrhosis is a common condition in Vietnam and worldwide. In 2020, approximately 1.5 billion people had issues related to cirrhosis and chronic liver disease. Cirrhosis can be caused by a variety of factors, including nonalcoholic fatty liver disease, viral hepatitis, alcohol, biliary diseases, and autoimmune liver diseases [1].

The global mortality rate due to cirrhosis is relatively high, with approximately 16 deaths per 100,000 people. In Asia, this rate ranges from 8 to 34 per 100,000 people [2]. In Vietnam, the estimated mortality rate among hospitalized cirrhosis patients is approximately 27.7%. Most of these deaths are associated with complications of cirrhosis, such as gastrointestinal bleeding, hepatorenal syndrome, hepatic encephalopathy, and spontaneous bacterial peritonitis [3].

Esophageal varices are the primary cause of upper‐gastrointestinal bleeding in patients with cirrhosis and represent the most common life‐threatening complication [4]. The mortality rate within the first 6 weeks following an initial gastrointestinal bleeding episode is approximately 15%–20% [5, 6]. Therefore, early detection of signs of portal hypertension and esophageal varices is crucial in diagnosing, predicting, and treating cirrhosis. Oesophagogastroduodenoscopy (EGD) is a highly valuable method for accurately diagnosing and classifying the severity of esophageal varices [7, 8, 9, 10, 11]. However, this is a high‐tech, invasive method, and performing regular endoscopies in specialized health care facilities poses challenges for patients. Globally, and in Vietnam, the value of noninvasive methods such as the APRI, PAP, platelet count, and liver elastography as key diagnostic factors are being explored, laying the groundwork for oesophagogastroduodenoscopy (EGD) to confirm the diagnosis of esophageal varices in cirrhosis patients [10, 12, 13, 14, 15, 16]. Numerous studies have shown that the levels of albumin, bilirubin, and platelet count are significant indicators for predicting esophageal varices in cirrhosis patients [15, 17, 18]. The ALBI and ALBI–PLT scores, which are calculated based on the parameters of albumin, bilirubin, and platelet count, have shown superior predictive ability for esophageal varices compared to individual indices alone [14, 19, 20, 21, 22].

Currently, there are studies worldwide that assess the value of the ALBI and ALBI–PLT scores in predicting and evaluating the severity of esophageal varices in patients with hepatocellular carcinoma and cirrhosis [19, 20]. In Vietnam, the ALBI and ALBI–PLT scores have been implemented and studied, with some reports focusing on patients with hepatocellular carcinoma. However, there are still limited data on the application of these scores in cirrhosis patients. Although several noninvasive models have been proposed to predict esophageal varices, most studies have been conducted in Western populations or in patients with hepatocellular carcinoma. Data evaluating the performance of ALBI‐based models in heterogeneous cirrhotic populations in Southeast Asia remains limited. Moreover, the clinical utility of these scores as triage tools for endoscopic screening in real‐world healthcare settings has not been well explored. Therefore, validating these models in different regional populations is essential before broader clinical application. Given the practical need to better understand the incidence of esophageal varices in cirrhosis patients and to evaluate the predictive value of the ALBI and ALBI–PLT scores for esophageal varices, we conducted this research to answer the following question: “Do the ALBI and ALBI–PLT scores have predictive value for esophageal varices in cirrhosis patients?” The findings from this study may lead to recommendations for the application of these scores in clinical practice. These scores are intended to serve as noninvasive triage tools rather than replacements for endoscopy, with the aim of optimizing screening strategies in real‐world clinical settings.

## 2 Materials and Methods

Over an 8‐month period (November 2022 to May 2023), consecutive patients with a clinical diagnosis of liver cirrhosis who were referred for screening endoscopy at the Gastroenterology Department of Cho Ray Hospital were prospectively enrolled. Cirrhosis was diagnosed based on clinical features, laboratory findings, and imaging results consistent with chronic liver disease.

All participants provided written informed consent before inclusion. The study protocol was approved by the Institutional Review Board of the University of Medicine and Pharmacy at Ho Chi Minh City. Patients were excluded if they had hepatocellular carcinoma, portal vein thrombosis, active gastrointestinal bleeding, prior endoscopic therapy for varices, or were receiving medications that might influence platelet counts or portal hypertension (e.g., non‐selective beta‐blockers). Patients with severe hepatic encephalopathy or coma were also excluded.

A comprehensive medical history was obtained, and clinical and general examinations were performed. Clinically, patients were classified using the updated Child–Turcotte–Pugh system. According to this system, Child A is assigned 5–6 points, Child B is assigned 7–9 points, and Child C is assigned 10–15 points [23]. The presence and grade of esophageal varices were evaluated using upper oesophagogastroduodenoscopy [24].

Tests for liver function, including total bilirubin, serum albumin, alanine transaminase (ALT), aspartate transaminase (AST), prothrombin time and activity, and the international normalized ratio (INR), were conducted. Renal function tests, covering urea and creatinine, along with a complete blood count and erythrocyte sedimentation rate (ESR), were also performed. Additionally, each patient underwent an abdominopelvic ultrasound to examine the liver, portal venous system, spleen, collateral vessels, and other abdominal organs.

The ALBI grade was calculated as follows: −0.085 × (albumin g/L) + 0.66 × log(bilirubin μmol/L). The ALBI grades were categorized as follows: ALBI I ≤ −2.60, ALBI II > −2.60 to ≤ −1.39 and ALBI III > −1.39 [21].

The ALBI–PLT score was calculated by adding the ALBI grade and points for platelet count (1 point for platelet count > 150,000/mm^3^ and 2 points for platelet count ≤ 150,000/mm^3^). The ALBI–PLT score ranges from 2 to 5 [21].

### 2.1 Statistical Analysis

Data were analyzed using Stata Statistical Software (Release 14.2; StataCorp LLC, College Station, TX, USA). Continuous variables are presented as mean ± standard deviation (SD) for approximately normally distributed data and as median (interquartile range [IQR]) for non‐normally distributed data. Categorical variables are presented as *n*/*N* (%). Group comparisons were performed using Student's *t* test for normally distributed continuous variables and the Mann–Whitney *U* test for non‐normally distributed continuous variables. Categorical variables were compared using the *χ* ^2^ test or Fisher's exact test, as appropriate. Receiver operating characteristic (ROC) analysis was used to evaluate discrimination, and the area under the curve (AUC) was reported with 95% confidence intervals (CI). The optimal cut‐off values were determined using the Youden index. Sensitivity, specificity, positive predictive value (PPV), and negative predictive value (NPV) were calculated with 95% CIs when applicable. All tests were two‐sided, and *p* < 0.05 was considered statistically significant.

## 3 Results

Table 1 shows the comparisons between patients with and without esophageal varices. Compared with patients without varices, patients with esophageal varices were the same age (56.16 ± 10.69 vs. 56.42 ± 10.90 years; *p* = 0.884).

**Table 1:** Baseline characteristics, investigations, and scores.

		Total (*n* = 152)	No EVs (*n* = 67)	EVs (*n* = 85)	*p*
Age (year)		56.31 ± 10.78	56.16 ± 10.69	56.42 ± 10.90	0.884^a^
Height (cm)		161.23 ± 10.41	161.54 ± 5.98	160.99 ± 12.89	0.748^a^
Weight (kg)		59.08 ± 10.78	57.48 ± 6,69	60.34 ± 13.04	0.104^a^
Sex	Female	52 (34.2%)	22 (32.8%)	30 (35.3%)	0.751^b^
	Male	100 (65.8%)	45 (67.2%)	55 (64.7%)	
Clinical ascites		44 (28.9%)	9 (13.4%)	35 (41.2%)	< 0.001^b^
Total bilirubin (mg/dL)		1.51 (2.16)	1.08 (1.16)	2.30 (2.09)	< 0.001^c^
Albumin (g/L)		35.48 ± 7.75	39.27 ± 7.29	32.48 ± 6.74	< 0.001^a^
AST (U/L)		43.5 (25.5)	39 (26)	47 (24)	0.019^c^
ALT (U/L)		29.5 (22.5)	31 (26)	27 (22)	0.077^c^
Hemoglobin (g/L)		114.13 ± 24.47	125.01 ± 23.09	105.54 ± 22.11	< 0.001^a^
Platelets (10^9^/L)		107.5 (95)	140 (145)	81 (77)	< 0.001^c^
INR		1.32 ± 0.32	1.21 ± 0.26	1.42 ± 0.34	< 0.001^a^
ALBI score		−2.06 ± 0.79	−2.48 ± 0.75	−1.73 ± 0.67	< 0.001^a^
ALBI–PLT grade	2	23 (15.1%)	20 (29.9%)	3 (3.5%)	< 0.001^b^
	3	37 (24.3%)	24 (35.8%)	13 (15.3%)	
	4	62 (40.8%)	17 (25.4%)	45 (52.9%)	
	5	30 (19.7%)	6 (8.9%)	24 (28.2%)	

*Note:* The data are presented as the means ± standard deviations, numbers (percentages) for nominal data and medians (interquartile ranges) for nonnormally distributed data.

Abbreviation: EVs, esophageal varices.

^a^ *t‐*test.

^b^ *χ* ^2^ test.

^c^ Mann–Whitney test.

Patients with esophageal varices had significantly (*p* < 0.05) greater (median [IQR]) serum total bilirubin (2.30 [2.09] vs. 1.08 [1.16] mg/dL), serum AST (47 [24] vs. 39 [26] U/L), and INR (1.42 ± 0.34 vs. 1.21 ± 0.26).

Moreover, they had significantly (*p* < 0.05) lower (median [IQR]) serum albumin concentrations (32.48 ± 6.74 vs. 39.27 ± 7.29 mg/dL), hemoglobin concentrations (105.54 ± 22.11 vs. 125.01 ± 23.09 g/dL), WBCs (4.45 [2] vs. 5.9 [3] mm^3^) and platelet counts (81 [77] vs. 140 [145] 10^9^/L).

**Figure 1:**
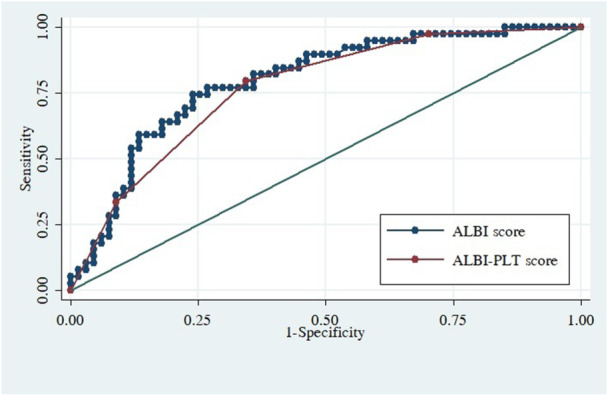
Receiver operating characteristic (ROC) curves showing the diagnostic performance of the ALBI and ALBI–PLT scores for predicting the presence of esophageal varices in patients with liver cirrhosis.

Compared with patients without varices, patients with esophageal varices (Table 1 and Figure 1) had significantly (*p* < 0.05) greater ALBI scores (−1.73 ± 0.67 vs. −2.48 ± 0.75).

ROC curve analysis was used to assess the usefulness of the ALBI score and the ALBI–PLT score as noninvasive models for detecting esophageal varices and discriminating their grade or size (Table 2).

**Table 2:** Receiver operating characteristic (ROC) curve analysis of the ALBI and ALBI–PLT scores in patients with and without esophageal varices and in patients with small vs. large varices.

	ALBI	ALBI–PLT
Varices detection		
AUC	0.778	0.763
*p*	< 0.001	< 0.001
95% CI	0.702–0.839	0.688–0.828
Cut‐off	> −2.76	> 2
Sensitivity	96.5%	96.5%
Specificity	41.8%	29.9%
PPV	67.8%	63.6%
NPV	90.3%	87.0%
Large varices detection		
AUC	0.794	0.769
*p*	< 0.001	< 0.001
95% CI	0.703–0.865	0.682–0.849
Cut‐off	> −1.89	> 3
Sensitivity	66.7%	97.4%
Specificity	77.6%	29.9%
PPV	63.4%	44.7%
NPV	80.0%	95.2%

Abbreviations: NPV, negative predictive value; PPV, positive predictive value.

For detecting varices of any size (Figure 1), an ALBI > −2.76 had 96.5% sensitivity, 41.8% specificity, 67.8% PPV, and 90.3% NPV. An ALBI–PLT score > 2 had 96.5% sensitivity, 29.9% specificity, 63.6% PPV, and 87.0% NPV. A pairwise comparison of the ROC curves for detecting esophageal varices of any size revealed that both the ALBI–PLT and ALBI were comparable (*p* = 0.561). Although the specificity of both scores was relatively low, their high sensitivity and NPV make them suitable as rule‐out rather than rule‐in tools. This pattern has also been reported in previous studies evaluating ALBI‐based models in cirrhotic populations. The relatively low specificity observed for both ALBI and ALBI–PLT scores in our study is consistent with findings reported in previous studies evaluating non‐invasive models for predicting esophageal varices. These models are generally designed to prioritize sensitivity and negative predictive value rather than specificity. In clinical practice, this approach is appropriate for screening purposes because the primary objective is to avoid missing patients with clinically significant varices.

**Figure 2:**
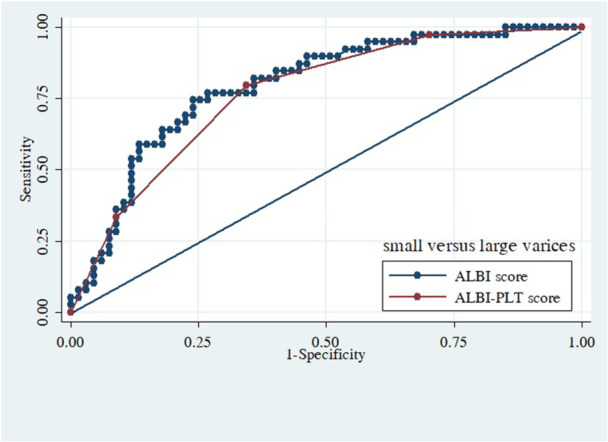
Receiver operating characteristic (ROC) curve analysis of the ALBI and ALBI–PLT scores in patients with small vs. large varices.

For the discrimination of large varices (Figure 2), an ALBI > −1.89 had 66.7% sensitivity, 77.6% specificity, 63.4% PPV, and 80.0% NPV. An ALBI–PLT score > 3 had 97.4% sensitivity, 29.9% specificity, 44.7% PPV, and 95.2% NPV. Pairwise comparison of ROC curves for the discrimination of large varices revealed that both the ALBI score and ALBI–PLT score were comparable (*p* = 0.400).

## 4 Discussion

EV dilation is a common complication in patients with cirrhosis, occurring in 50% of patients with cirrhosis [25]. The annual incidence of EVs increases by approximately 7%–8% for patients with cirrhosis, with 10%–12% of small varices progressing to large varices. The prevalence of EVs is dependent on the stage of cirrhosis, with a prevalence rate of 40% at Child–Turcotte–Pugh (CTP) stage A and up to 85% at CTP stage C [26]. Upper‐gastrointestinal bleeding due to EV rupture is a severe complication that occurs in approximately 30% of patients with cirrhosis. Despite significant advancements in diagnosis and treatment, the mortality rate from upper‐gastrointestinal bleeding caused by ruptured varices remains high, at approximately 20% [27].

Oesophagogastroduodenoscopy (EGD) is the gold standard for diagnosing and screening EVs in patients with cirrhosis. However, the cost of screening and monitoring EVs using EGD is relatively high. EGD is an invasive procedure that can cause significant discomfort and inconvenience for patients, and not all patients consent to frequent endoscopies [17]. Therefore, current trends among researchers involve the use of noninvasive methods to predict the presence of EVs and monitor the progression of existing varices. This approach is used with the aims of reducing the need for endoscopy, decreasing health care costs, and improving patient compliance and satisfaction.

Among our patients with esophageal varices, total bilirubin levels were significantly greater, and albumin levels were significantly lower (both *p* values < 0.001). This can be attributed to the fact that patients with esophageal varices generally have a higher Child–Pugh class, indicating more severe cirrhosis, and increased bilirubin is a marker of declining liver function. This observation aligns with findings reported by Tiwari et al. [28] and Alsebaey et al. [20].

In a large‐scale international study, Johnson et al. [29] evaluated liver function in patients with hepatocellular carcinoma to explore its relationship with survival rates following treatment. They introduced the albumin–bilirubin score (ALBI), a measure for assessing liver dysfunction. Unlike the CTP, the ALBI score is straightforward, less subjective, and uses only two routine laboratory tests. This score also demonstrated a correlation with survival. Several publications have investigated the ALBI score in the context of hepatocellular carcinoma, covering its application from diagnosis to treatment [30, 31]. The success of these methods has encouraged researchers to assess them in other liver diseases. A 2016 study in China by Zou et al. involving 381 cirrhosis patients revealed that an ALBI score with a cut‐off point greater than −1.49 had a sensitivity of 100%, a specificity of 69.6%, a PPV of 7.4%, and an NPV of 100% in predicting in‐hospital mortality due to upper‐gastrointestinal bleeding in cirrhotic patients. The AUC for this prediction was 0.87 (95% confidence interval: 0.84–0.91) [22].

The ALBI score was substantially different between the studied groups and could be used as a predictor for esophageal varices in our research. At a cut‐off point of −2.76, the *p* value was < 0.001, the sensitivity was 96.5%, the specificity was 41.8%, the PPV was 67.8%, and the NPV was 90.3%. Alsebaey et al. also reported that an ALBI cut‐off value of −2.43 served as a reliable predictor for esophageal varices, demonstrating a sensitivity of 81.28%, a specificity of 74.89%, a PPV of 58.9%, and an NPV of 90% [20].

The ALBI score, with a cut‐off point of −1.89, exhibited a sensitivity of 66.7%, a specificity of 77.6%, a PPV of 40%, and an NPV of 52% when used to detect large‐sized esophageal varices. This finding has been validated in two studies. Chen et al. [21], in a study of patients with hepatocellular carcinoma (HCC), reported that the sensitivity of the ALBI score for predicting high‐risk esophageal varices in patients with compensated HCC was 96.0%, with a specificity of 27.1%, a PPV of 21.1%, and an NPV of 97.1%. Alsebaey et al. [20] reported that an ALBI greater than −1.88 had a sensitivity of 92.9%, a specificity of 60.6%, a PPV of 55.9%, and an NPV of 94.1% for detecting large esophageal varices.

The ALBI–PLT score can serve as a reliable predictor for the presence of esophageal varices in patients with compensated cirrhosis, providing a simple and noninvasive means to identify patients at low risk for developing esophageal varices, thus reducing the need for unnecessary endoscopic screening. A retrospective and prospective study by Chen et al., published in 2018 in Taiwan, examined 1530 patients, approximately 70% of whom had compensated cirrhosis. The prevalence of EVs was 37.8%. Using an ALBI–PLT score cut‐off of > 2, the study reported a sensitivity of 90%, specificity of 27%, PPV of 21%, and NPV of 97% for predicting large varices. The authors concluded that patients with an ALBI–PLT score ≤ 2 should not undergo EGD to check for varices, as it could save costs and avoid unnecessary invasive procedures [21].

Additionally, Miyamoto et al. reported a significant correlation between the ALBI–PLT score and endoscopic findings of gastroesophageal varices (GEVs). They observed that the incidence of GEVs increased with increasing ALBI–PLT, and the likelihood of high‐risk varices also increased with increasing score [32].

In our study, the ALBI–PLT score was found to be a predictor of the presence of esophageal varices at a cut‐off point greater than 2, with a sensitivity of 96.5%, a specificity of 29.9%, an NPV of 87%, and a PPV of 63.6%. Additionally, with a sensitivity of 97.4%, a specificity of 29.9%, an NPV of 95.2%, and a PPV of 44.7%, a cut‐off point greater than 3 could be used as an indicator for large esophageal varices. It should be noted that the parameters used in the ALBI and ALBI–PLT scores may be influenced by several systemic factors. Serum albumin levels can be affected by nutritional status, systemic inflammation, and renal dysfunction, while bilirubin levels may vary in conditions such as acute hepatitis or hemolysis. Platelet counts may also be influenced by infection or hypersplenism. To minimize potential confounding effects, patients with acute complications or conditions known to significantly alter these parameters were excluded from the study. Nevertheless, these laboratory markers also reflect the overall severity of liver dysfunction, which is closely associated with portal hypertension and the development of esophageal varices.

The results of a study by Alsebaey et al. [20] align with our findings, showing that an ALBI–PLT score of 3 had a sensitivity of 77.3%, a specificity of 72.9%, a PPV of 55.9%, and an NPV of 87.9%. Similarly, with an ALBI–PLT score greater than 4, they reported a sensitivity of 39.44%, a specificity of 75%, a PPV of 45.9%, and an NPV of 69.7%.

The ALBI–PLT cut‐off in our study was lower than that in another study, with values of 2–3 versus 3–4 [20]. This difference can be attributed to the choice of cut‐off by the authors in the other study, which had much lower sensitivity (77.3% vs. 96.5%) but considerably higher specificity (72.9% vs. 29.9%). This suggests that while their approach aimed to reduce false positives, it could result in a greater number of false negatives.

Importantly, the ALBI and ALBI–PLT scores are not intended to replace endoscopic screening. Instead, they should be considered as initial triage tools that may help identify low‐risk cirrhotic patients who could safely defer immediate screening endoscopy. This approach may be particularly useful in resource‐limited healthcare systems where universal endoscopic screening is not always feasible. Several limitations should be acknowledged. First, this was a single‐center study with a relatively modest sample size, which may limit the generalizability of the findings. Second, ALBI and ALBI–PLT scores were evaluated at a single time point, and longitudinal changes in these scores were not assessed. Third, external validation in an independent cohort was not performed. Therefore, larger multicenter studies are required to confirm the reproducibility and clinical utility of these findings.

## 5 Conclusion

The ALBI and ALBI–PLT scores are useful noninvasive triage tools for predicting the presence of esophageal varices and may help identify low‐risk patients who could safely defer screening endoscopy, particularly in settings with limited resources.

## Author Contributions

**Thang Dinh Nguyen:** conceptualization, methodology, writing – original draft, validation, formal analysis, data curation. **Huong Tu Lam:** data curation, formal analysis. **Thong Duy Vo:** conceptualization, investigation, writing – original draft, methodology, validation, visualization, writing – review and editing, supervision.

## Funding

The authors have nothing to report.

## Ethics Statement

The study was performed following the ethical guidelines of the Declaration of Helsinki (6th revision, 2008). The research was approved by the Research Ethical Committee of University of Medicine and Pharmacy at Ho Chi Minh City, Viet Nam, with approval number 812/HDDD‐DHYD.

## Consent

Written informed consent was obtained from all participants prior to enrollment.

## Conflicts of Interest

The authors declare no conflicts of interest.

## Transparency Statement

The corresponding author (Thong Duy Vo) affirms that this manuscript is an honest, accurate, and transparent account of the study being reported; that no important aspects of the study have been omitted; and that any discrepancies from the study as planned (and, if relevant, registered) have been explained.

## Data Availability

Raw data were generated at Cho Ray Hospital. Derived data supporting the findings of this study are available from the corresponding author on request.
